# Hepatocellular carcinoma: old friends and new tricks

**DOI:** 10.1038/s12276-020-00527-1

**Published:** 2020-12-02

**Authors:** Eunsun Kim, Patrick Viatour

**Affiliations:** 1 Stanford Cancer Institute, Stanford University, Stanford, CA USA; 2 Children’s Hospital of Philadelphia, Center for Childhood Cancer Research, Philadelphia, PA USA; 3 Department of Pathology and Laboratory Medicine, Perelman School of Medicine, University of Pennsylvania, Philadelphia, PA USA

**Keywords:** Liver cancer, Tumour immunology, Cancer genomics

## Abstract

Hepatocellular carcinoma (HCC) is the most prevalent primary liver cancer and a leading cause of cancer-related deaths worldwide. Ninety percent of HCC cases arise from cirrhosis, during which liver cells undergo chronic cycles of necrosis and regeneration. The complex genomic landscape of HCC has been extensively investigated to draw correlations between recurrently mutated pathways and patient prognosis. However, our limited success with targeted therapy shows that knowing the presence of somatic mutations alone is insufficient for us to gauge the full spectrum of their functional consequences in the context of tumor evolution. In addition, the current molecular classification of HCC offers little information on the relationship between the molecular features and immunological properties of HCC tumors and their immune microenvironment. This review introduces current challenges and advancements made in HCC surveillance, diagnosis, and treatment. We also discuss the suite of HCC-associated genetic changes and describe recent studies that provide evidence for an evolving functional model and its implications for understanding and targeting HCC progression.

## Liver cancer: Call to explore evolving mutations within tumors

Hepatocellular carcinoma (HCC) is the most common primary liver cancer worldwide, but despite advances in understanding disease genetics, successful targeted therapies are limited. Eunsun Kim at Stanford University and Patrick Viatour at the University of Pennsylvania reviewed current HCC surveillance, diagnostics and treatments, focusing on genetic changes and their influence on disease progression. Novel methods for HCC diagnosis, including analysis of cancer-derived materials in blood, will complement existing diagnostics and help identify more early stage cases. Extensive studies of HCC tumor ecosystems will inform the development of novel immunotherapies. More studies involving genetic sequencing of mutations within tumors over time should further advance our understanding of HCC progression and help explain varying treatment responses.

## Introduction

Hepatocellular carcinoma (HCC) accounts for 90% of primary liver cancer, which is the fourth most common cause of cancer-related deaths worldwide^1,2^. The HCC incidence rate associated with nonalcoholic fatty liver disease (NAFLD) and nonalcoholic steatohepatitis (NASH) is increasing, and the global burden of mortality from HCC is predicted to reach 1 million deaths annually by 2030^3,4^. HCC is asymptomatic in its early stage, which significantly delays its timely diagnosis. Those diagnosed at the advanced stage of disease are ineligible for curative surgery, and therapeutic options for advanced HCC patients are limited in availability and efficacy^5^. To improve the current state of early diagnosis and treatment, we need a better understanding of HCC. Here, we examine recent shifts in the epidemiology and etiology of HCC as the socioeconomical status, age structure, and dietary patterns of the global population change. At the forefront of HCC research are antiviral therapies against hepatitis C virus (HCV), liquid biopsy of circulating tumor cells and circulating tumor DNA, biomarkers for early surveillance and diagnosis, and targeted therapies using a novel mechanism of action^6,7^. We discuss these efforts as well as the ongoing investigation of the HCC immune microenvironment, which is critical for us to understand the molecular mechanism behind immunotherapy for HCC. Last, this review introduces paradigm-shifting study results on the different roles that somatic mutations play in the context of HCC progression. All of these novel findings are expected to facilitate the development of more efficient treatments by advancing our understanding of HCC.

## Epidemiology of HCC

The mortality and incidence rates of HCC vary by geographical region and socioeconomic status. Primary liver cancer is most prevalent in sub-Saharan Africa and Eastern/South-Eastern Asia^8^. In males, the regions of high incidence are Eastern Asia, with 26.8 age-standardized incidence rates (ASIRs, per 100,000), and South-Eastern Asia (21.0). The regions of intermediate incidence rates are Europe (6.2–10.9) and Northern America (10.1), and those of low incidence rates are Western Asia (5.4) and South-Central Asia (3.4). In females, the regions of high incidence rates are Melanesia (8.9) and Eastern Asia (8.7), and that of a low incidence rate is South-Central Asia (1.7). It should be noted that values reported by GLOBOCAN are the combined incidences of HCC and cancers of intrahepatic bile ducts, the latter of which are disproportionately concentrated in Eastern/South-Eastern Asia but rare in Europe and North America^9^. The overall male-to-female ratio for age-standardized liver cancer incidence and mortality is ~2.4. Studies suggest that estrogen prevents and androgen promotes HCC^10^, and estrogen-mediated inhibition of IL-6 has long been suggested as a mechanism by which estrogen exerts its protective effect against HCC^11^. A more recent work proposed that reduced adiponectin secretion in males during puberty may be responsible for the higher liver cancer risk in males^12^.

Previous studies have used the expression “developed” or “less-developed” to draw a negative correlation between HCC incidence and the economic status of a country or region. However, these terms are vague and do not fully reflect the comprehensive socioeconomical status of a region. In 2015, the Global Burden of Disease (GBD) introduced the Socio-Demographic Index (SDI) as a standardized method of measuring the sociodemographic development of a country by incorporating data on average income per person, educational attainment, and total fertility rate (TFR)^13^. The GBD Liver Cancer Study from 2015 showed that 88% of new liver cancer cases and 86% of liver cancer deaths occurred in middle-SDI, high-middle SDI, and high-SDI countries compared to low- or low-middle-SDI countries^4^. The ASIR was highest in high-income Pacific Asia (Brunei, Japan, South Korea, and Singapore) (26.4), with Japan showing a gradual decrease in HCC cases. The HCC incidence rate decreased by 20% in Eastern and Western sub-Saharan Africa, and within the Chinese populations in Hong Kong, Shanghai, and Singapore, where hepatitis B virus (HBV) vaccination and hepatitis C virus (HCV) antiviral treatment programs became more accessible. Conversely, there has been an increase in the ASIR in high-SDI countries such as the United States, Canada, Australia, and Northern European countries, mostly due to a shift in the population age structure and changes in dietary patterns. This global trend is projected to continue through 2030^14–16^.

## Etiology of HCC

In most cases, HCC develops with underlying cirrhosis and chronic liver inflammation^17,18^. The major risk factors for HCC are HBV/HCV infection, heavy alcohol consumption, aflatoxin B1 ingestion, tobacco smoking, and nonalcoholic fatty liver disease (NAFLD) caused by obesity and insulin resistance^17,19^. Although the contribution of different etiologies to liver cancer deaths varies by region, HBV is the leading cause of global liver cancer incidences and deaths (272,000 and 265,000 cases, respectively)^4^. Approximately 3.5% of the world’s population (257 million) was infected with HBV in 2015, with the highest prevalence (percentage of infected individuals within the population) in African (6.1%) and Western Pacific (6.2%) regions and the lowest prevalence in the Americas (0.7%)^20^. There are two major ways that HBV can be transmitted: vertical (perinatal; from an infected mother to her newborn) and horizontal (open cuts, blood transfusions, sexual transmissions, etc.). The most effective method of controlling HBV transmission is universal infant vaccination of birth dose (HepB_BD) followed by three doses of HBV vaccination (HepB3) within the first year of life^21^. Due to the increased availability of HBV vaccines, the estimated prevalence of HBV infection in children <5 years of age (during which HBV prevention is most effective) has decreased from 4.7% to 1.3%, with the exception of those in select African regions where birth dose vaccination coverage is unavailable^22^. Successful outcomes of nationwide vaccination programs implemented by Taiwan and Italy in 1984 and 1987, respectively, proved that HBV infection and HCC incidence rates can be controlled by a rigorous vaccination program^23,24^. Within 10 years of the program launch, the overall prevalence rate of hepatitis B surface antigen (HBsAg) drastically decreased in both countries, accompanied by a declining trend in liver cancer mortality. This shows that an efficient vaccination program can prevent liver cancer from HBV infection.

HCV affects ~1% of the world population (71 million), with the highest prevalence in the Eastern Mediterranean (2.3%) and European (1.5%) regions and the lowest prevalence in South-Eastern Asia (0.5%). Vaccines for HCV do not exist, but antiviral therapies have been effective in reducing and suppressing viral loads in HCV-infected individuals^25^. Infection is considered eradicated when there is a sustained virologic response (SVR), defined as the absence of detectable HCV RNA 24 weeks after the end of therapy. Prior to 2014, the standard of care for chronic HCV patients was interferon-based peginterferon-ribavirin (PEG-IFN/RBV) therapy, with an SVR of <50%^26^. In 2016, the World Health Organization (WHO) updated its guidelines, and direct-acting antiviral agents (DAAs) replaced IFN therapy for screening and treating HCV-infected individuals^27,28^. DAAs (NS3/4A protease inhibitors, NS5B nucleos(t)idic and non-nucleos(t)idic polymerase inhibitors, and NS5A replication complex inhibitors) target HCV-specific enzymes essential for viral replication. First-generation DAAs (telaprevir and boceprevir) were administered in combination with PEG-IFN/RBV therapy and achieved a significantly higher SVR (IFN + telaprevir: 75%, IFN + boceprevir: 68%) than IFN-based therapy alone (40–44%)^29,30^. The most recent development in HCV treatment was IFN-free pangenotypic DAAs, which combine 2 or 3 second-generation DAAs (e.g., glecaprevir-pibrentasvir, sofosbuvir-daclatasvir, and sofosbuvir-velpatasvir). Compared to IFN therapy, which shows a range of SVRs depending on the HCV genotype, pangenotypic DAAs achieve an overall higher treatment efficacy against all six major HCV genotypes, with a > 90% SVR and reduced risk for HCC^31^.

Environmental factors can also increase the risk for HCC. Although alcohol itself is not mutagenic, heavy alcohol consumption increases the risk for the development of cirrhosis. Individuals with cirrhosis due to HCV and alcohol consumption are at a significantly higher risk of HCC than those with cirrhosis due to alcohol consumption alone^32^. Dietary exposure to aflatoxin B1, a mycotoxin produced by the *Aspergillus* fungus, is most common in Asia and the tropical regions of Africa. Aflatoxin B1 is often ingested with contaminated grains and metabolized by hepatic cytochrome p450. Once metabolized, it acts as a DNA mutagen that induces the transversion of G →T in the third position of codon 249 in *TP53*. HCC patient populations exposed to aflatoxin B1 and HBV tend to overlap, and some studies suggest that there is a strong synergistic association between the two^33^. Smoking tobacco contributes to HCC initiation by accelerating liver fibrosis, and long-term usage of tobacco is closely associated with HCC^34–36^. A recent US-based prospective cohort study of 1,518,741 individuals (“Liver Cancer Pooling Project”) investigated the association between smoking and HCC and showed that smoking is associated with a 55% increased risk for HCC^37^. Smoking introduces hepatocarcinogens (e.g., acetylaminofluorene and 4-aminobiphenyl) into the body, which increases the production of proinflammatory cytokines and induces oxidative stress, triggering lipid peroxidation^38^. When combined with preexisting conditions such as HBV/HCV infection, chronic alcohol usage, and obesity, the long-term consequences of tobacco smoking can be severe.

Last, NAFLD is a spectrum of chronic liver diseases that exhibit excessive cytoplasmic retention of triglycerides in >5% of hepatocytes. NAFLD occurs in the absence of excessive alcohol consumption and is strongly associated with metabolic diseases such as obesity, insulin resistance, and type 2 diabetes. NAFLD affects 25% of the global population, with the highest prevalence in the Middle East and South America and the lowest prevalence in Africa^39^. The NAFLD incidence rate is also rising in industrialized countries such as the United States as adult obesity increases^40^. NAFLD can range from simple steatosis to nonalcoholic steatohepatitis (NASH), which can progress into liver fibrosis and cirrhosis^3^. NAFLD and NASH are associated with an increased risk for HCC, and patients with cryptogenic cirrhosis are at a high risk for developing HCC. According to White et al.^41^, who performed a meta-analysis of 17 independent cohort studies, 18 case-control and cross-sectional studies, and 26 case series, NAFLD or NASH cohorts with few or no cases of cirrhosis cases had a minimal risk for HCC (cumulative HCC mortality of 0–3% for study periods up to 20 years) than cohorts with cirrhosis. Accordingly, the current standard of clinical practice recommends routine HCC screening in patients with NAFLD-related cirrhosis and excludes patients without cirrhosis, although some studies suggest that patients with NAFLD can develop HCC in the absence of cirrhosis^42,43^. Limitations in study design and analysis (i.e., small cohort size, insufficient information on patient risk factors, and short follow-up period) that may contribute to these conflicting outcomes are discussed in a study by Torres et al., where most recent clinical studies on NAFLD and HCC are reviewed in detail^44^.

## Surveillance and diagnosis of HCC

Alpha fetoprotein (AFP) is the most widely used biomarker for HCC surveillance and diagnosis. It is the only phase V biomarker available for HCC surveillance and is used in combination with ultrasound scanning (USS), multiphasic computed tomography (CT), and magnetic resonance imaging (MRI)^45^. However, AFP assessment has poor sensitivity (47–64%) for detecting HCC and is ineffective in accurately detecting early HCC^46^. Osteopontin, Midkine, and GALAD are phase III biomarkers awaiting further validation^47^. GALAD is a statistical model that can diagnose HCC and predict patient survival by incorporating parameters such as sex, age, and the levels of AFP, AFP-L3, and des-γ-carboxyprothrombin (DCP) from peripheral blood. In a study of three independent cohorts (Germany, Japan, and Hong Kong), GALAD demonstrated higher sensitivity and specificity in HCC diagnosis than AFP, AFP-L3, or DCP alone and is currently in clinical trials as a new HCC surveillance tool^48^. If approved, it could be effective for monitoring high-risk individuals. Other potential biomarkers of early HCC include heat shock protein 70 (HSP70), cyclase-associated protein 2 (CAP2), glypican-3 (GPC3), glutamine synthetase (GS), and Golgi protein 73 (GP73), but none has been clinically approved to date^49–52^.

Liquid biopsy is an attractive alternative to traditional tissue biopsy for cancer diagnosis and prognosis^7,53^. It is minimally invasive, better reflects the heterogeneous mutational spectrum of the tumor, and allows multiple specimen collections for monitoring tumor evolution in high-risk individuals. Liquid biopsy specimens include circulating tumor cells (CTCs), circulating tumor DNA (ctDNA), cell-free microRNA, and extracellular RNA. Among these, CTCs and ctDNA are the most actively investigated. CTCs are cancer cells shed by primary tumors into the blood circulation^54^. Epithelial cell adhesion molecule (EpCAM) is commonly used as a surface marker for detecting CTCs in various cancers, including HCC. However, only a fraction of HCC tumors express EpCAM, which suggests that EpCAM-dependent assays can introduce bias towards EpCAM^+^ HCC and may not be suitable for predicting all HCCs^55^. Studies have reported that the presence of CTCs in HCC patients is associated with tumor growth, invasiveness, recurrence and metastasis, but most of these studies were based on a small patient population and lacked prospective long-term follow-up data^56–58^.

CtDNA is fragmented circulating free DNA (cfDNA) released by apoptotic and necrotic tumor cells into the circulation^59^. The active release of ctDNA by living tumor cells has also been reported, but the mechanism is unclear. ctDNA is identified by sequencing for tumor-specific mutations such as somatic mutations, methylation changes, the integration of viral DNA, the loss of heterozygosity, and chromosomal aberrations^60,61^. In particular, tumor-specific ctDNA methylation has been shown to outperform AFP in sensitivity and specificity as both a diagnostic and prognostic marker for HCC^62^. However, for utilization in early surveillance, the ctDNA sequencing method has to be highly sensitive to detect low ctDNA levels in asymptomatic individuals and would drive up the cost of cancer screening^63^. Future steps for ctDNA detection analysis include the development of tissue-specific assay panels to trace the origin of ctDNA, a more sensitive ctDNA isolation method to overcome the current limitations of a low ctDNA detection rate in patient blood, and a more efficient sequencing platform to lower the cost of ctDNA analysis.

## Treatment strategies for HCC

Surgical resection, liver transplantation, transarterial embolization (TACE), radiofrequency ablation, and systemic therapy can extend patient life expectancy^64^. For early stage HCC patients with no history of cirrhosis or portal hypertension, resection is recommended, but it is associated with a recurrence rate of ~60–70% at 5 years, prompting repeated resection and further treatment^65^. Early stage HCC patients with liver dysfunction can benefit from liver transplantation, which has a 4-year survival rate of >80% under the Milan criteria (﻿single tumor ≤5 cm or three tumors all ≤3 cm at the time of transplantation)^66^. However, early HCC is clinically asymptomatic, and small nodules (<2 cm) are difficult to characterize by a radiologic or pathologic examination. Delays in cancer detection contribute to the high death rate of HCC patients, which was exemplified by the ratio of estimated deaths (~30,000) versus newly diagnosed cases of HCC (~40,000) in 2018^67^. Patients with intermediate HCC and intact liver function are eligible for transarterial embolization or radiotherapy, which is used to prevent the progression of unresectable tumors. However, whether either treatment can prolong survival in the absence of systemic chemotherapy remains controversial and requires further validation.

Treatment for advanced-stage HCC patients is limited to systemic therapy. Systemic therapy includes standard cytotoxic chemotherapy (e.g., FOLFOX4 regimen: fluorouracil, leucovorin, oxaliplatin), which is not routinely used in HCC patients, targeted (chemo)therapy such as sorafenib, and immunotherapy^68^. Sorafenib is a multikinase small molecule inhibitor that targets vascular endothelial growth factor receptors (VEGFR) 1–3, platelet-derived growth factor receptor-β (PGRFR-β), and rapidly accelerated fibrosarcoma (Raf) family kinases. For almost a decade, sorafenib was the only first-line systemic targeted drug available for advanced HCC, with a survival benefit of 3 months. All phase III trials of new targeted therapies failed to show a survival benefit, whether as a stand-alone drug or in combination with sorafenib^69^. Between 2017 and 2019, new first-line and second-line systemic therapies emerged, all targeting tyrosine kinase receptors: regorafenib (a multikinase inhibitor using a similar mechanism of action as sorafenib)^70^, lenvatinib (a multikinase inhibitor targeting VEGFR 1–3, fibroblast growth factor receptors (FGFR) 1–4, PDGFR α, RET, and KIT)^71^, cabozantinib (a tyrosine kinase inhibitor targeting MET, VEGFR2 and RET)^72^, and ramucirumab (a VEGFR2 antagonist)^73,74^.

Immunotherapy is the latest development in HCC treatment. The majority of HCCs arise from chronic liver disease, in which T cells are constantly exposed to antigen and inflammatory signals. This condition induces a state of T-cell exhaustion during which T cells lose their effector functions and upregulate inhibitory receptors such as programmed cell death protein-1 (PD-1), cytotoxic T-lymphocyte-associated protein 4 (CTLA-4), and T-cell immunoglobulin mucin-3 (TIM-3)^75,76^. This suggests that HCC patients could benefit from reversing T-cell exhaustion and inhibiting tumor cell immune escape through immunotherapy. Immune checkpoint inhibitors and adoptive chimeric antigen receptor (CAR) T-cell therapy are the two pillars of immunotherapy. Each works through a different mechanism; immune checkpoint inhibitors enhance the T-cell response by blocking inhibitory pathways that prevent effective antitumor T-cell responses, whereas adoptive CAR T-cell therapy introduces an immune effector that specifically targets cancer cells. Nivolumab is a PD-1 inhibitor that was approved in 2017 as a second-line treatment for advanced HCC^77^. In 2020, the combination of nivolumab and ipilimumab (a monoclonal antibody targeting CTLA-4) was approved for treating advanced-stage HCC patients who were on or intolerant to sorafenib^78^. PD-1/PD-L1 inhibitors are currently being tested together with receptor tyrosine kinase inhibitors to increase the efficacy of treatment, with some showing promising results. Patients with unresectable HCC were treated with the combination of atezolizumab (targeting PD-L1) and bevacizumab (a monoclonal antibody targeting VEGF) and showed better overall (67.2% at 12 months vs. 54.6%) and progression-free (6.8 months vs. 4.3 months) survival than those treated with sorafenib alone^79^.

Achieving a clinical response using CAR T cells requires the presence and identification of appropriate tumor-associated antigens (TAAs). This is particularly challenging in solid tumors such as HCC because solid tumor antigens are often intracellular or secreted. There is currently no CAR T-cell therapy available for HCC patients, but preclinical study results look encouraging, as the adoptive transfer of CAR T-cells targeting AFP and GPC3 was shown to reduce tumor burden in mouse xenograft models of liver cancer^80,81^.

## Genomic landscape and molecular classification of HCC

Over the past two decades, studies have utilized next-generation sequencing (NGS) strategies to explore the genomic landscape of HCC^19,82,83^. Recurrent mutations (defined as a mutational frequency of >5%) in HCC are associated with the following pathways: telomere maintenance, Wnt/β-catenin, TP53/cell cycle, oxidative stress response, epigenetic regulation and chromatic remodeling, and PI3K/AKT/mTOR and Ras/Raf/MAPK^19,82–84^. The conventional “genomic landscape of HCC” is largely based on the somatic mutations of a tumor from a specific timepoint. Although informative, this “snap-shot” approach provides limited insight into the functional consequences of the mutations that arise during the sequential evolution of HCC from cirrhosis. It also does not address whether and how intratumoral somatic mutations affect the tumor immune microenvironment. More recently, there has been an increased effort to reconstruct the genomic landscape of HCC. Some of the noteworthy studies are introduced in this section.

### Overview of recurrently mutated pathways in HCC

Telomerase reactivation enables cancer cells to bypass checkpoint signaling pathways that are activated upon telomere shortening and overcome replicative senescence^85,86^. Telomerase reverse transcriptase (TERT) is a catalytic subunit of the enzyme telomerase, and *TERT* promoter mutation reactivates telomerase. *TERT* promoter mutation is nearly absent in cirrhotic livers, but its frequency drastically increases during the malignant transformation from dysplastic nodules to HCC (6% of LGDNs, 19% of HGDNs, 61% of early HCCs, and 42% of small and progressed HCCs)^87^. *TERT* promoter mutation is recognized as the most frequent (43–64%) and earliest alteration in HCC^88^.

The Wnt/β-catenin pathway is a key developmental pathway that regulates liver homeostasis and zonation^89,90^. Approximately 15–33% of HCC patients carry activating mutations in β-catenin (*CTNNB1*)^91,92^, and 17% carry inactivating mutations in Axin1 (*AXIN1;* 11–15%) or adenomatous polyposis coli (*APC;* 1–2%)^19,83,93^. Mutations targeting these components prevent β-catenin degradation, which leads to aberrant Wnt signaling activation.

p53 is a tumor suppressor that regulates cell cycle arrest, apoptosis, and senescence in response to cellular stress. *TP53* inactivating mutations (12–48%) are mostly found in the DNA binding domain, with the R249S mutation in HCC linked to aflatoxin B1 and HBV infection^94,95^. *TP53* mutations are associated with high chromosomal instability and rarely cooccur with *CTNNB1* mutations^19^. Other cell cycle pathway mutations include the homozygous deletion of cyclin-dependent kinase inhibitor 2A (*CDKN2A;* 2–9%), HBV insertion in cyclin E1 (*CCNE1;* 5%), and somatic mutations (i.e., homozygous deletions, truncating mutations, and missense mutations) in retinoblastoma 1 (*RB1;* 3-8%). Although mutations in the *RB1* gene itself occur at a relatively low frequency, at least one component of the RB pathway is altered in 80–92% of human HCCs, resulting in unrestricted E2f activity. These alterations include p16^INK4A^ family inactivation by hypermethylation or homozygous deletion (40–70%), cyclin D (*CCND1)* overexpression by *CCND1* amplification or by upstream oncogenes (22–58%), or gankyrin overexpression^96–101^.

Chronic liver inflammation can induce an oxidative stress response, producing an excess of reactive oxygen species (ROS) and provoking DNA damage and genomic instability. The oxidative stress pathway is activated in 5–14% of HCCs, primarily by activating mutations in nuclear factor erythroid 2-related factor 2 (Nrf2, encoded by *NFE2L2;* 3–6%) or inactivating mutations in kelch-like ECH-associated protein 1 (*KEAP1;* 2–8%)^83,84^. Nrf2 is a transcription factor that regulates the expression of cytoprotective genes in response to xenobiotic and oxidative stress^102^. Keap1 is an adapter protein for an E3 ubiquitin ligase complex that negatively regulates Nrf2 expression by marking it for degradation and preventing its entry into the nucleus. Constitutive activation of the Nrf2/Keap1 pathway due to the defective proteasomal degradation of Nrf2 protects cancer cells from ROS toxicity and promotes HCC tumor growth.

The SWItch/Sucrose Non-Fermentable **(**SWI/SNF) chromatin remodeling complex regulates DNA histone interactions in an ATP-dependent manner^103^. SWI/SNF complex genes exhibit strong tissue specificity, and specific mutations are enriched in different cancers. AT-rich interaction domain 1A *(ARID1A)* and ARID2 are core components of two separate subunits of SWI/SNF complexes (BAF and PBAF, respectively), and they are recurrently mutated in HCC (5–15% and 3–15%, respectively)^92^. *ARID1A* mutations are significantly associated with *CTNNB1* mutations and alcohol-induced HCC^19^, and *ARID2* mutations cooccur with *NFE2L* mutations^104^.

Activating mutations of *PIK3CA* (1.6–2%) and inactivating mutations of tuberous sclerosis 1 (*TSC1)* or *TSC2* (3–8%), genes involved in the phosphatidylinositol-3-kinase (PI3K)/Akt/mammalian target of rapamycin (mTOR) signaling pathway, are found in HCC. Recurrent inactivating mutations in ribosomal protein S6 kinase A3 (*RPS6KA3;* 9.6%), a gene involved in the rat sarcoma virus (Ras)/rapidly accelerated fibrosarcoma (Raf)/mitogen-activated protein kinase (MAPK) pathway (also called Ras/Raf/MEK/ERK pathway), have also been identified, especially in noncirrhotic HCC. *RPS6KA3* encodes ribosomal S6 protein kinase 2 (RSK2), a kinase member of the Ras/MAPK signaling pathway that is directly activated by extracellular-regulated kinases 1 and 2 (ERK1/2). RSK2 is a negative regulator of Ras signaling, and the inactivation of RSK2 by *RPS6KA3* mutations leads to constitutive activation of the Ras pathway. Other components of the PI3K and Ras pathways are rarely mutated (<2%)^19,83^.

### Recurrent mutations in the context of HCC progression

Recurrent mutations in HCC are often categorized as “early” or “late” despite the inconsistency between the timing of the mutations and their functional outcome. A recent sequencing study showed that a majority of somatic mutations in key driver genes are present throughout disease progression^104^. Most mutations are evenly distributed from early to advanced HCC, with the exception of *TP53* mutations, which occur more frequently in advanced HCC. In addition, the “classic genetic drivers” (e.g., *TERT* promoter, *AXIN1, ARID1A*, and *ARID2*) do not show significantly different mutation frequencies between early and advanced HCC. The study results support earlier findings from Kim et al. and Sun et al., where key somatic mutations in *CTNNB1* and *ARID1A* occurred in early HCC and persisted throughout the course of the disease^105,106^^.^ Both studies used mouse models of HCC to show that *CTNNB1* and *ARID1A* play different roles in the context of HCC progression, independent of the presence of the mutation.

*CTNNB1* is thought to be a dominant oncogenic driver of HCC through its role in Wnt signaling activation^107–109^. However, the transgenic expression of constitutively active β-catenin in the liver is not sufficient to initiate HCC^110^, and the loss of β-catenin in chemically induced models of HCC paradoxically increases tumor progression^111,112^. These findings suggest that β-catenin may not be an oncogenic driver, which brings to question the functional relevance of its high mutational frequency in HCC. Our recent work, Kim et al.^105^, provided evidence that β-catenin is not a dominant oncogenic driver of HCC but rather a tumor promoter with complex and evolving functions. We showed that β-catenin is predominantly located at the membrane of HCC cells, where its association with cadherin family members supports EGFR pro-survival signaling, independent of the Wnt pathway mutational status. We found that Wnt pathway mutations occur as early as stage I HCC and that their mutational frequencies persist throughout the course of the disease, but the nuclear translocation of β-catenin (which signifies Wnt signaling activation) is restricted to the advanced stage. This suggests that Wnt pathway mutations may have other discrete tumor-promoting functions that sustain tumor growth in the early stage. These lesser-known tumor-promoting functions may be related to immune evasion, as tumor cells with active β-catenin signaling have been shown to downregulate cytokine production and impair dendritic cell recruitment to escape T-cell infiltration in melanoma and HCC^113,114^. In addition, Wnt pathway activating mutations in patients treated with immune checkpoint inhibitors are associated with a low disease control rate and short median overall survival time^115^.

*ARID1A* is generally known as a tumor suppressor, and its loss is associated with increased migration, invasion, and metastasis. However, Sun et al.^106^ showed that ARID1A has two opposing functions that are dependent on the timeline of HCC progression and that *ARID1A* promotes tumor initiation during the early stage by increasing the oxidative stress response but suppresses tumor metastasis and invasion in the late stage. *ARID1A* mutation is an early event in HCC, but reduced ARID1A protein expression is predominantly found in advanced or metastatic HCC^104^. This suggests that early *ARID1A* mutations may have a more discrete function in supporting early HCC—similar to *CTNNB1* mutations— and further work is required to investigate the mechanism.

### Exploring the HCC immune microenvironment using a single-cell sequencing platform

The single-cell RNA sequencing (scRNA-seq) platform allows the real-time assessment of transcriptomic changes in a specific cell population at the single-cell level. It is a powerful tool for analyzing cancers with highly heterogeneous transcriptomes, such as HCC, where subtle but functionally relevant transcriptional differences may not be obtained through bulk approaches. A number of scRNA-seq studies on nonparenchymal cell and immune cell populations in normal or diseased livers have been published in the last 3–5 years.

The fibrotic niche of human liver cirrhosis and the interactome of cell lineages that contribute to cirrhosis are largely unknown. Ramachandran et al.^116^ recognized this gap in knowledge and offered a detailed characterization of multiple nonparenchymal cell populations in fibrotic patient livers. The same study also identified scar-associated cell markers and pathogenic subpopulations of macrophages, endothelial cells, and collagen-producing myofibroblasts that may have clinical implications.

The current topic of interest in the liver cancer field is why HCC patients show a low response rate to immunotherapy (15–20% objective response rate)^77^. To address this question, Zhang et al.^117^ combined two dominant scRNA-seq platforms (10x and SMART-seq2) to study CD45^+^ cells isolated from HCC tumors and four immune-relevant sites (adjacent liver, hepatic LNs, blood, and ascites) and showed potential migration patterns of tumor-infiltrating immune cells.

Ma et al.^118^ used scRNA-seq analysis to probe another critical question: “whether and how the HCC tumor transcriptome affects its immune microenvironment.” The study showed that HCC tumors with highly diverse transcriptomes expressed significantly higher levels of *VEGFA* than those with less diverse transcriptomes and that diverse transcriptomes are associated with aggressiveness and a poor prognosis. The pathways expressed in CD8^+^ and CD4^+^ T cells from highly diverse HCC are different from those expressed in T cells from less diverse HCC (EMT and myogenesis versus oxidative phosphorylation and interferon-α (IFN-α)/IFN-γ, respectively), which suggests that the HCC tumor transcriptome may indeed affect its immune microenvironment. This study provided a potential explanation for the clinical outcomes observed in PD-1-treated HCC patients. Their findings opened doors to future functional experiments on the mechanism of action behind the combined therapy of receptor tyrosine kinase inhibitors and immune checkpoint inhibitors in advanced HCC patients.

### Updates on the molecular classification of HCC

Efforts to map the association between the mutational landscape of HCC and patient survival have resulted in various HCC classification methods. Lee et al.^119^ analyzed global gene expression patterns in HCC patients to show two distinct molecular subclasses of HCC (A and B) that can predict patient survival. Boyault et al.^120^ stratified HCC into 6 subgroups (G1-G6) based on the HCC transcriptome, clinical features (i.e., HCC risk factors), genetic and epigenetic alterations, and chromosomal instability^93^. Hoshida et al.^121^ combined gene expression datasets from eight independent cohorts to identify three major subclasses of HCC (S1-3) based on clinical phenotypes and the activation of specific molecular pathways (mainly Wnt, TGF-β, Myc, and p53). Last, Villanueva et al.^122^ used genome-wide methylation profiling to report 36-probe methylation signatures that can predict patient survival across different stages of HCC and further performed an integrative analysis on transcriptome data to identify key oncogenes and tumor suppressor genes with aberrant methylation. Based on the aforementioned classifications, HCC can be regrouped into two major molecular classes (“proliferative” and “non-proliferative”); the difference between the two subclasses is described in detail in Zucman-Rossi et al.^84^. Although all of these classification methods provide valuable insight into HCC, they offer limited information on the relationship between the molecular features and clinicopathological properties of the disease as HCC progresses from an early to advanced stage.

The following recent papers addressed these limitations by utilizing the clinical and multiomics database from The Cancer Genome Atlas (TCGA): Sia et al. and Shimada et al. categorized HCC based on cancer etiology, recurrence patterns, and immunological features and validated their findings against the TCGA dataset^123–126^. Their updated HCC classification method identifies a subgroup of HCC with gene signatures related to immune cell infiltration and could help predict patients who may benefit from immune checkpoint therapy. Nault et al.^104^ performed whole-exome sequencing of HCC tumors from early to advanced stages to determine the correlation between HCC tumor stage and recurrently mutated pathways and compared their findings to the TCGA genomic sequencing dataset. They showed that recurrent mutations are found in all tumor stages, except for *SETD2* mutations, which are not found in early HCC. They also reported that different molecular subgroups of the proliferative and non-proliferative classes were found in all clinical stages but in a different distribution. This suggests that there may be a time-linearity element to the molecular subclasses that was previously overlooked.

## Conclusion

The epidemiology and etiology of HCC have changed in the last 30 years, as have the ways we prevent, diagnose, and treat HCC. While HCV vaccines do not exist, DAA-based HCV therapies are successful in reducing viral loads in infected individuals, reaching an SVR or >90% and decreasing mortality associated with HCC. Novel methods of HCC surveillance and diagnosis, such as GALAD or tcDNA liquid biopsy, are under development or in clinical trials. In particular, tcDNA methylation analysis shows much promise as an early diagnostic and prognostic tool, with higher sensitivity and accuracy than AFP. Most of these methods are not intended to completely replace current methods of surveillance and diagnosis, as they themselves have technical limitations, and their cost-benefit relationship must be taken into account. Instead, they can complement existing methods to maximize reliability and efficiency.

HCC immunotherapy holds great promise but also faces many challenges. Currently, there is no standardized way to predict patient responsiveness to immune checkpoint inhibitors because the consequences of intertumoral mutations on cancer-driven immune evasion are largely unknown^127^. Why immune checkpoint inhibitors work for some HCC patients but not for others is an incredibly complex question that requires us to re-examine HCC as a tumor ecosystem that embodies tumor cells, immune cells, endothelial cells, stromal cells, and nonmalignant cells in a highly dynamic relationship with one another^128^.

NGS is an indispensable discovery tool for molecular research, and liver tumors have been aggressively sequenced from whole tissue to single-cell levels using various methods (e.g., whole genome, exome, RNA) across multiple cohorts. We are now at a point where the genomic landscape of HCC has already been identified, and further sequencing of HCC tumors must be accompanied by a clear hypothesis-driven objective to advance our understanding of the disease. More than ever, we need to pay close attention to the functional consequences of intratumoral mutations in the context of HCC progression. The most recent work by Ma et al.^129^ is a model example of a hypothesis-driven scRNA-seq study featuring tumor, nonparenchymal, and immune cell populations from multi-timepoint biopsies to capture the transcriptomic changes that occur in the tumor ecosystem as patients undergo immunotherapy. We predict that more studies will follow suit and adopt a comprehensive approach to characterize HCC tumors and their responses to different treatments at various time points of cancer progression. By doing so, we will be able to better predict the course of the disease and provide appropriate targeted therapies to patients.
